# Citation Metrics: A Primer on How (Not) to Normalize

**DOI:** 10.1371/journal.pbio.1002542

**Published:** 2016-09-06

**Authors:** John P. A. Ioannidis, Kevin Boyack, Paul F. Wouters

**Affiliations:** 1 Meta-Research Innovation Center at Stanford (METRICS), Stanford University, Stanford, California, United States of America; 2 Stanford Prevention Research Center, Department of Medicine, Stanford University School of Medicine, Stanford, California, United States of America; 3 Department of Health Research and Policy, Stanford University School of Medicine, Stanford, California, United States of America; 4 Department of Statistics, Stanford University School of Humanities and Sciences, Stanford, California, United States of America; 5 SciTech Strategies, Inc., Albuquerque, New Mexico, United States of America; 6 Centre for Science and Technology Studies, Leiden University, Leiden, Netherlands

## Abstract

Citation metrics are increasingly used to appraise published research. One challenge is whether and how to normalize these metrics to account for differences across scientific fields, age (year of publication), type of document, database coverage, and other factors. We discuss the pros and cons for normalizations using different approaches. Additional challenges emerge when citation metrics need to be combined across multiple papers to appraise the corpus of scientists, institutions, journals, or countries, as well as when trying to attribute credit in multiauthored papers. Different citation metrics may offer complementary insights, but one should carefully consider the assumptions that underlie their calculation.

## Abstract 2

Citation metrics are very influential and their normalization is a contentious issue. Each normalization approach has advantages and disadvantages that need to be understood for proper use of these metrics.

Citation metrics have proliferated over the years. This rapidly growing literature is aiming to find the most fair and unbiased approach to appraise papers, scientists, institutions, and journals. Thus, citation metrics can have major implications that affect the entire scientific community and are not of interest just to experts in bibliometrics. Their use and misuse cause controversies not only for technical reasons but also for emotional reasons because these metrics judge scientific careers, rewards, and reputations [1]. Scientists, journals, or institutions scoring badly in specific metrics may hate them and those scoring well may love them.

A core question about citation indicators is whether and how to normalize them. The basic premise of normalization is that not all citations are equal. Therefore, normalization can be seen as a process of benchmarking that is needed to enhance comparability across diverse scientists, fields, papers, time periods, and so forth. The term “rescaling” is also used instead of “normalization” in some disciplines, such as physics and computational sciences [2–4].

One can focus first on normalizing citations received by single papers, since a single paper is the smallest unit for which normalization can be considered. This offers a good way to illustrate the main concepts and challenges involved. However, normalization issues can be expanded from the single paper to the assessment of larger units of published work including many papers. These collections of papers may pertain to whole scientific fields or subfields, CVs of single scientists, teams of scientists, and institutions, nations, and journals. In particular, there is a very extensive literature on normalized journal-level metrics, such as the Source Normalized Impact Per Paper (SNIP). Journal-level metrics are a hot field because of the longstanding debate about whether any valid inferences can be made from them to “rank” journals. Their detailed discussion goes beyond the scope of the current paper.

Article-level citation counts tend to have very long-tailed distributions, with many articles getting no or few citations and a few articles getting a large number of citations. For papers in the long tail, it is sometimes difficult to say what constitutes a large enough difference (e.g., whether 50 citations received in two years is meaningfully different from 30 citations received in the same time frame). The long tails are generated by a preferential attachment process, in which some articles attract more and more citations. Citations mean that the work has attracted interest, but the exact reasons why it has attracted interest may vary for different papers.

Ideally, one wants citation indicators to measure impact in a monotonic fashion: the higher the metric, the “better” the paper. Citations received by a single paper may depend on multiple factors beyond pure merit. These include the scientific field where the paper belongs (different fields have different numbers of publishing and citing scientists and citing cultures, and thus different citation density), the age (how long ago it was published), the type of document (article, review, letter, editorial), and the coverage of the database where citations are counted.

Scientific field normalization sounds intuitive [5]. One wants to correct for imbalance of citation opportunity. Other things being equal, an influential paper in a theoretical mathematics field where reference lists are traditionally short may attract fewer citations than influential papers in fields where papers typically have much longer reference lists [6]. Moreover, sometimes the fact that citations are higher in one field than in another may simply reflect that the research in the former field is, in a certain sense, more important scientifically and more fruitful than the research in the latter. After all, it is implausible that all scientific fields contribute equally to the stock of scientific knowledge and share an equal proportion of great scientists among their investigators. The scientific workforce and, even more so, great scientists are particularly attracted to specific disciplines and research questions that change over time. The widely used Web of Science classification of fields is based on classifying journals in subject categories, and these subject categories may be very unequal in importance, newsworthiness, or genuine breadth of appeal. E.g., in Web of Science, the subject category “Medicine, General and Internal” includes all major medical journals; these journals justifiably receive far more citations than journals in specialty categories. Also, innovative “sleeping beauty” papers may be minimally cited until their results are recognized by the scientific community. Furthermore, the very criteria for truth and excellence are not historical constants but evolve as part of science and scholarship [7].

A major challenge is how to define scientific fields for normalization. Fields have been categorized in the past on the basis of journals or library categories. Within-field citations are usually denser than between-field citations. However, no field is isolated, and between-field communication is increasingly common nowadays [8]. In some areas, the boundaries between fields seem to become less distinct. Different categorizations may segregate science to anywhere between a dozen [9] to several thousands of disciplines or specialties [10]. Fields may be defined a priori (e.g., based on the journals where research gets published) or dynamically (e.g., based on citing or cited papers or on the references of citing or cited papers) [11]. The rationale is that citing papers consider cited papers relevant to their work, so they belong to the same field. Obviously, this is not true for all citations—e.g., some methods (statistical, laboratory, or other) can be used by multiple unrelated fields, and there are also substantive interdisciplinary references.

When fields are very broad, they spuriously conglomerate many subfields with very different citation densities and/or importance. Conversely, when fields are very specific, normalization is based on a few reference papers and has a large error margin. Moreover, narrow fields may vary enormously in their contribution to the advancement of knowledge. Normalizations using fields defined from citing or cited papers may lead also to counterintuitive situations; e.g., when a paper starts being cited in another, remote field, this may signify it has acquired extra importance. However, if that paper is cited by another field that has high citation density, its normalized citation score may decrease rather than increase, depending on the precise construction of the normalization procedure [12]. Overall, there is some evidence that definition of fields based on cocitation, such as the approach recently proposed by the relative citation ratio (RCR) method [13], is better than using taxonomies using journal categories. Alternative approaches to define fields that use direct citation and bibliographic coupling have been proposed to be even better [14], but the verdict is not final. The superiority of one approach over others may depend on the time frame, the database, and types of articles used. The extent to which limitations of different approaches are frequent or not requires further empirical evaluation [8,15]. For example, in an assessment of 200,000 articles published between 2003 and 2010, only 0.2% experienced a drop in RCR of 0.1 [13].

Age (year of publication) seems a straightforward normalizing factor. A paper published in 2000 has had more time to accrue citations than one published in 2015. Therefore, each paper can be compared against papers published in the same year. However, with an acceleration in the number of scientific papers published annually, influential old papers had a far smaller literature that could have cited them within a few years of publication as compared with more recent influential papers. Moreover, scientific fields (no matter how defined) don’t make the same progress each and every year. There are good and bad years. A paper in the top 10% of citations published in a year of major progress may be a more fundamental knowledge contribution than a paper in the top 1% of a year when the field stagnated. Finally, calendar year–based normalization produces noisy results in very recent papers. These are the papers for which the most rigorous appraisal of impact is desirable, since they reflect the recent or current dynamism of a scientist or institution. A paper published in January 2015 has had 14 months to be cited until March 2016, while a paper published in December 2015 has had only 3 months. Many journals also use advance online posting, so a paper published in 2015 might have been available to cite since 2013.

Normalization for type of document also poses several challenges. Review articles receive more citations than articles with new empirical data, and some types of papers (such as letters or editorials) often receive few or no citations [16,17]. A first challenge is to identify accurately the different types of document categories. Besides misclassification errors (i.e., reviews tagged as articles with new data and vice versa [18]), each document category can include article types with different connotations and citation profiles. “Reviews” may include nonsystematic expert assessments, systematic reviews, meta-analyses, prospective meta-analyses, collaborative consortia analyses, etc. These differ enormously in inherent credibility, scientific value, amount of work required, contribution to science, and reasons for being cited. Many high-quality “reviews” are more important in all these aspects than the vast majority of uninformative “original research.” Penalizing these reviews would be inappropriate; their higher citations reflect their higher value. Conversely, many expert reviews propagate unfounded opinions in large citation networks; their numerous citations may simply measure how they distort science [19]. Letters and editorials are often easier to identify accurately in databases and are more homogeneous in getting few citations. Therefore, one may exclude them in assessing citation indicators. Nevertheless, occasionally timely editorials may contribute more to scientific progress than “original” papers. Disruptively innovative ideas may be easier to express in non–peer-reviewed editorials than in peer-reviewed research.

Citation database coverage may affect all of the factors discussed above. Detailed discussion of citation databases is beyond our scope here. Briefly, databases have variable coverage of different scientific fields, years, and types of documents, and coverage may change over time [20]. Errors and transparency on included document sources also differ. Among the most popular databases (Web of Science, Google Scholar, and Scopus), Web of Science has the longest historical coverage. Both Web of Science and Scopus have higher transparency and data quality but lower overall coverage than Google Scholar and prominent deficiencies in social sciences, humanities, and many other fields. Depending on the goal of the bibliometric analysis, more complete coverage may be desirable, but it needs to be consistent, otherwise the inclusion of irrelevant items (e.g., trade journals) may complicate the delineation of fields and proper normalization of indicators. Besides database coverage, differences may also exist in preprint culture across fields. Some fields, such as economics, have protracted peer review, and many papers are available as preprints; thus, a large share of citations are made to preprints. Conversely, molecular biology has had no preprint culture to date (although this may change in the future with efforts such as BioRxiv).

Multiple other contentious factors may be considered as options to model in the normalization process. Should citations matter more when they come from journals and/or papers that score more highly themselves in citation metrics? This may promote a self-reinforcing effect. Text mining approaches may also allow giving different weight to citations based on the context in which they are made, how often they are mentioned in the citing paper, and whether they are favorable or critical of the work being cited [21,22]. However, text mining requires full texts (often nonaccessible), and it is unclear whether one can easily separate generation of fruitful debate (even if the citing authors disagree) from renunciation of error. Negative citations tend to be relatively rare [23], except in some of the social sciences, where disputes may be played out by citing each other (rather than by ignoring one another). Some controversy exists also on how to handle self-citations. Table 1 summarizes factors that may be considered or debated during normalization and shows for comparison two systems, the RCR method [13] and the normalization used in the Leiden Ranking of universities (http://www.leidenranking.com), both of which use citation data from the Web of Science.

**Table 1:** Factors that have been considered in normalization of citation metrics and their application in two normalization systems.*

	*Relative citation ratio*	*Leiden system*
Scientific field definition	Defined by network of citing papers	3,822 micro-level fields based on citations of all papers
Scientific field fixed or dynamic	Dynamic, different for each cited paper	Each paper is assigned to one micro-field
Scientific field broad or narrow	Can vary a lot	Mostly moderate size
Age (year of publication)	Accounted for	Accounted for
Type of documents	Multiple types	Articles and reviews
Citing sources	Not adjusted for	Not adjusted for
Place of citations in citing sources	Not adjusted for	Not adjusted for
Multiplicity of reference in citing source	Not adjusted for	Not adjusted for
Context of citation in citing source (supportive versus negative or critical)	Not adjusted for	Not adjusted for

*This does not mean necessarily that normalization for these factors improves the validity of the citation results.

Additional challenges emerge when extending to the assessment of multiple papers—e.g., the published corpus of a scientist, institution, or country. For example, how should the (normalized) metrics of single papers be summarized and interpreted? Multiple options exist (Table 2).

**Table 2:** Some options for summarizing and interpreting (normalized) citation metrics from single papers across multiple papers.

Averaging ratios of actual citations versus expected citations for each paper
Ratio of sum of actual citations divided by sum of expected citations
Proportion of papers in top 1% of normalization or other reference group
Proportion of papers in top 10% of normalization or other reference group
Proportion of papers in top 50% of normalization or other reference group
Other combinations of percentile ranks of multiple papers—e.g., R(6), R(100), R(6,k), R(100,k)

For example, it can also make a difference on whether one focuses on extremely highly cited papers, top 1%, top 10%, the average paper, or other parts of the distribution. Do we prefer an institution or scientists producing papers that are mostly above the average but none that have extremely high impact, or those producing papers mostly at the average or even below but that occasionally come up with an extremely high-impact publication? Normalization is most unstable at the extremes, where few papers compete. It would be silly to try to normalize the *Origin of Species* versus the description of the DNA helix (32,556 and 11,551 citations per Google Scholar, respectively, as of February 2, 2016) to see which one is better.

Another major confounding issue is the allocation of credit. An increasing number of papers are authored by many authors. Given the large extent of multiauthorship in most scientific fields [24,25], adjustments for multiauthorship and author contributions may have a much larger impact on citation indicators for scientists than other types of normalization and/or correction. Unfortunately, the exact contributions of authors are rarely disclosed, disclosures may be inaccurate, and even when accurate they are not easy to translate unambiguously into a quantitative equivalent. Author order is a widely used surrogate of contribution, with greater contributions typically attributed to first and last and/or corresponding authors. However, alphabetic author ordering is used in some fields [26,27]. There is an array of different quantitative approaches on how to correct for author order and multiauthorship [28–30].

Given the large volume of papers on variously normalized citation metrics, one may falsely infer that citation metrics are so confusing that they should be abandoned in favor of appraisal by experts in the field. Yet, citation metrics are so widely visible that no expert can close their eyes to them anyhow, even if he or she wanted to do so. Scientometrics may help put this type of information into perspective and understand the strengths and limitations of each metric in each setting and allow for diversity and plurality of career paths. Metrics and normalizations should be seen in the context of their assumptions. When results and conclusions are similar with different assumptions, this is reassuring. Conversely, when conclusions differ, one has to examine why and what the different assumptions signify. Finally, some metrics may be better suited than others in particular applications. E.g., different metrics and normalizations may make more sense in trying to identify top researchers versus finding out whether an institution is above or below average. Judicious use of citation metrics can still be very useful, especially when they are robust, transparent, and their limitations are properly recognized.

## Abbreviations

RCR: relative citation ratio
SNIP: Source Normalized Impact Per Paper
